# Participant experiences and engagement with the enhanced heart failure care program: a qualitative study

**DOI:** 10.1080/21642850.2026.2654919

**Published:** 2026-04-06

**Authors:** Hannah Fraser Wetzler, Andrew Boyle, Breanne Hobden, Aaron L Sverdlov, Nicholas Zwar, Rob Hungerford, Nicholas Collins, Trent Williams, Francesco Paolucci, Cameron Robson, Kristy Fakes

**Affiliations:** a School of Medicine and Public Health, College of Health, Medicine and Wellbeing, University of Newcastle, Callaghan, New South Wales, Australia.; b Hunter Medical Research Institute, New South Wales, Australia; c John Hunter Hospital, Hunter New England Local Health District, New South Wales, Australia; d Faculty of Health Sciences and Medicine, Bond University, Queensland, Australia; e School of Nursing and Midwifery, College of Health, Medicine and Wellbeing, University of Newcastle, Callaghan, New South Wales, Australia; f Newcastle Business School, University of Newcastle, Callaghan, New South Wales, Australia; g Department of Sociology, Law and Economics, University of Bologna, Bologna, Italy

**Keywords:** Heart failure, qualitative interview, depression, quality of life, internet-based intervention, randomised controlled trial

## Abstract

**Introduction:**

Mental health disorders are common among people with heart failure (HF). The Enhanced HF Care program is a digital health intervention designed to remotely monitor and support the wellbeing of patients discharged with acute decompensated HF via online modules and surveys. This qualitative study explores participants’ experiences, engagement with and feedback on the program.

**Methods:**

The design was a qualitative in-trial sub-study. Participants were recruited pre-discharge from two public hospitals in New South Wales, Australia. Semi-structured telephone interviews were conducted one- and/or six-months post-recruitment with a sample of participants who received the intervention to understand their experience of the program. Transcripts were analysed based on a qualitative descriptive approach and content analysis.

**Results:**

Twenty-four interviews were conducted with 18 participants (3 female, 15 male; age range 39–87 years). Four overarching themes were identified: program engagement; program benefits; additional information needs; and multidimensional perspectives on health. Most participants reported the program as beneficial, comprehensive and detailed enough for their needs, particularly valuing the opportunity for self‑reflection on mental and physical health. However, engagement was variable, with competing priorities and feeling overwhelmed after discharge limiting uptake for some participants. Easy post‑discharge access to information and the ability to share it with family were also valued.

**Discussion:**

Patient feedback highlighted the acceptability and positive impacts of the program, though some challenges remain for uptake including information overload and competing time priorities. These findings underscore the importance of timing, tailoring, and responsiveness to individual patient contexts within digital health interventions.

## 1. Introduction

Heart failure (HF) affects an estimated 64 million people worldwide (Savarese et al., 2022). Considerable morbidity and mortality, decreased quality of life and high healthcare and societal costs is attributed to HF. Acute decompensated heart failure (ADHF) refers to the worsening of symptoms from HF. It is a frequent cause of hospitalisation and is associated with high rates of mortality (Mebazaa et al., 2015). Poor mental health is common among patients with HF. Nearly half of patients with HF experience depressive symptoms, and about one-third exhibit symptoms of anxiety (Easton et al., 2016). Depression among patients with HF has been linked to increased hospital visits, diminished health functioning, and a higher risk of all-cause mortality (Lum et al., 2016). Diagnosing depression in patients with HF can be difficult due to an overlap between psychiatric and cardiac symptoms (Celano et al., 2018). Data indicate that providers do not recognise symptoms of depression in 50% of HF cases (Warraich et al., 2018). Several studies have also identified gaps in patient care for emotional wellbeing that, if addressed, could have positive outcomes for patients, families and health services (Harris et al., 2021; Hestevik et al., 2019; Lum et al., 2016).

Given the connection between emotional wellbeing and physical health outcomes (including HF progression) (Harris et al., 2021), a comprehensive approach to HF recovery is essential, which focuses on mental wellbeing, behavioural risk factors, and adherence to treatment (Fakes et al., 2024).

Contemporary HF guidelines recommend multidisciplinary management programmes with tailored education/self-care, post-discharge follow-up, and, where appropriate, telephone support/telemonitoring; exercise-based cardiac rehabilitation is recommended for all able patients (with supervised cardiac rehabilitation for higher-risk) (McDonagh et al., 2021). Given the high burden of depression and other psychosocial barriers in HF, guidelines also advise routine screening and referral, supporting digitally delivered education and remote check-ins (Heidenreich and Bozkurt, 2022; Levine et al., 2021).

In line with those recommendations, the Enhanced HF Care programme (henceforth referred to as ‘the programme’) was developed as a digital health intervention for patients discharged following a hospital admission with ADHF. The programme is being trialled to determine its effectiveness of remote monitoring and management of emotional and physical wellbeing to improve patients’ emotional and physical outcomes and in decreasing healthcare utilisation. The intervention is based on evidence that digital health interventions may be effective in helping patients with HF (Fakes et al., 2024; Kaihara et al., 2022).

A qualitative sub-study was conducted to gather participants' feedback on the programme. Qualitative enquiry is a well-established approach to inform health service delivery (Patton, 2014), including understanding the care experiences of patients with HF (Negarandeh et al., 2020; Thornhill et al., 2008; Vanneste et al., 2024). Whilst the Enhanced HF Care trial evaluates clinical effectiveness, evidence on patient experience of the programme, including perceived benefits, barriers and facilitators to engagement, may provide valuable complementary insights. To address this, a qualitative study was conducted to obtain in-depth insights into patients’ experiences and engagement with the programme.

### 1.1. Aim

To explore participants’ experiences, engagement with and feedback on the Enhanced HF Care Programme.

## 2. Methods

### 2.1. Study design

The study used a qualitative design, nested within the larger randomised controlled trial. Semi-structured telephone interviews were conducted to inform the interpretation of trial outcomes with a sample of participants from the programme.

Ethics approval for this study was obtained from the Hunter New England Human Research /ETH00502) and registered with the University of Newcastle Human Research Ethics Committee (H-2022–0227). The study was conducted in accordance with the Declaration of Helsinki and was approved by an Institutional Review Board/Ethics committee. The trial was registered with the Australian New Zealand Clinical Trials Registry (ANZCTR): 12622001289707.

This study adhered to the Consolidated Criteria for Reporting Qualitative Research (COREQ) (Tong et al., 2007).

### 2.2. Recruitment

Participants were recruited from two public hospitals in the Hunter New England Local Health District in New South Wales, Australia. Hospital 1 was a metropolitan centre with a regional and rural referral base, and Hospital 2 was a rural hospital.

Eligible patients were aged 18 years or older, able to read and speak English, and were considered physically and mentally capable of participating in the study by clinic staff.

For this qualitative component, a purposive sample of participants allocated to the intervention group were invited to complete up to two follow-up interviews at one- and/or six-months post-recruitment to explore their experiences and engagement with the online programme. Participants were selected based on time since recruitment.

#### 2.2.1. The online programme: enhanced HF care

**Figure 1.**
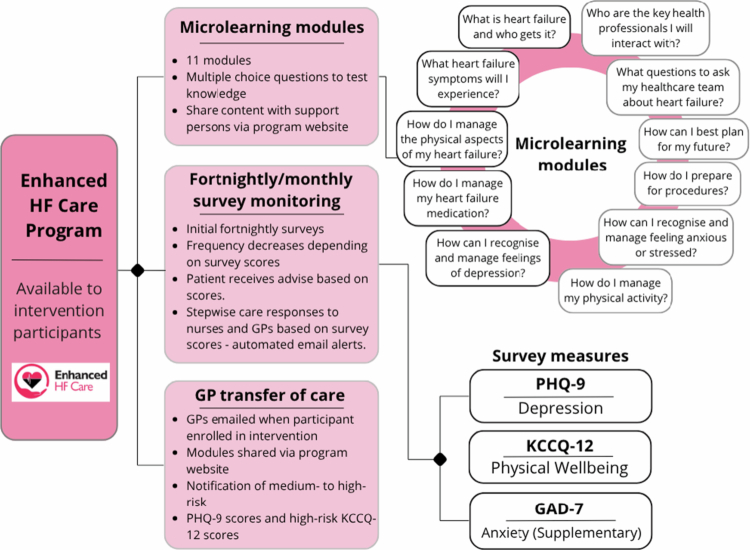
Summary of the Enhanced HF Care programme. Diagram demonstrating the three components of the Enhanced HF Care Programme. The first component shows microlearning module components, and demonstrates the 11 microlearning modules. The second component shows the fortnightly/monthly surveys participants, which are made up of 3 survey measures: PHQ-9, KCCQ-12 and GAD-7. The third component is a GP transfer of care letter.

A full description of the programme is outlined in the published protocol (Fakes et al., 2024). Briefly, the online programme includes a series of micro-learning modules that contain short videos and multiple-choice questions. All intervention participants were instructed to complete five essential modules, which comprised core information and actions for the post-discharge period. Six additional modules covering other topics were also offered (see Figure 1). Participants were encouraged to share the online learning modules with their support person/s or general practitioner (GP).

The online programme also included fortnightly/monthly surveys that monitored depression (Patient Health Questionnaire (PHQ)), anxiety (Generalised Anxiety Disorder (GAD)) and physical wellbeing (Kansas City Cardiomyopathy Questionnaire (KCCQ-12) with tailored self-care advice. If patients reported high levels of depression or clinical symptoms, cardiac nurses and participants’ GPs received automated email alerts with follow-up action.

### 2.3. Data collection

Telephone interviews were conducted with the purposive sample of participants. A telephone interview was deemed most appropriate, as it enabled participants to complete the interviews at a time and place that were convenient for them. Interviews took place at one- or six-months post recruitment, with some participants interviewed at both timepoints. This was done to ensure that both immediate impacts and longer-term reflections were obtained.

An experienced female researcher (HFW—undergraduate with honours) telephoned participants after written consent was obtained and invited them to complete an interview. The interviewer was neither involved in recruiting participants nor part of the patient care team.

Interviews followed a semi-structured interview guide with open-ended questions, designed to understand participant experiences with the programme, including ease of use, relevance and quality. The interview guide was designed to be flexible, with questions amended based on the participants’ responses. In addition, the length of each interview was determined by the individual participant. Before the interviews commenced, participants were advised that the telephone interviews were recorded, and that they could stop the interview at any time without giving a reason and could request at any time during the interview that any comments they made be erased from the recording.

### 2.4. Data analysis

A qualitative description approach was employed to identify similar themes in the data, expressed in the participants’ own language. This research approach was chosen for its suitability in gaining firsthand knowledge of patient experiences (Neergaard et al., 2009).

Interviews were recorded and transcribed verbatim. The qualitative analysis programme NVivo was used to code the transcripts, using thematic analysis. Initial codes were established based on the interview script questions (for example, ‘programme access’, ‘programme benefits’). Existing codes were refined, and additional codes were created as themes emerged throughout the transcripts. After the initial coding, each transcript was reviewed a second time and further coded to ensure consistency. The coding process was reviewed by KF, who also reanalysed a sample of the data. This author had detailed knowledge of the study and experience in conducting psychosocial and qualitative research.

Data saturation was reached when no new concepts emerged in interviews.

## 3. Results

Recruitment for the Enhanced HF Care Study commenced in . and , the research team contacted participants for interview until saturation of themes was reached. In total, 31 participants were contacted, out of 44 enroled in the intervention at that time.

Of those participants, seven declined to be interviewed, and six did not respond to the interview request. Eighteen individuals were interviewed, with four interviewed twice (at one month and six months). Overall, 24 interviews were conducted: 14 at one-month post-recruitment, and 10 at six-months post-recruitment. Interviews ranged from 3 to 36 minutes, with an average of 10 minutes.

Of the 18 participants interviewed, most were men (83%). The age range was 39 to 87 years old, with a median age of 65 years. Half of all participants (*n* = 9) were living with a spouse/partner at the time of interview, while a third were living alone (*n* = 6). Demographic data is presented in Table 1.

Two-thirds of participants were admitted to the study via Hospital 1, with the remaining third from Hospital 2. This is consistent with overall study site numbers. Half of all participants lived in Inner or Outer Regional Australia (*n* = 6 and *n* = 3, respectively).

**Table 1.** Participant Demographic Data (*n* = 18).

Characteristics		n
Sex	Female	3
Male	15	
Age (years)	Age range	39−87
	Mean Age	64
Highest level of education completed	High school (Year 7−12)	10
	Trade or vocational education (e.g. TAFE)	5
	University or post-graduate qualification	2
	Missing	1
Current marital status	Single, never married	2
	Married or living with partner	9
	Divorced or separated	4
	Widowed	2
	Missing	1
Lives with most of the time	With family members (such as partner, children, siblings)	11
	On my own	6
	Missing	1
Private health insurance	Yes	3
	No	14
	Missing	1
Remoteness	Major City of Australia	7
	Inner Regional	6
	Outer Regional	3
	Missing	2

**Figure 2.**
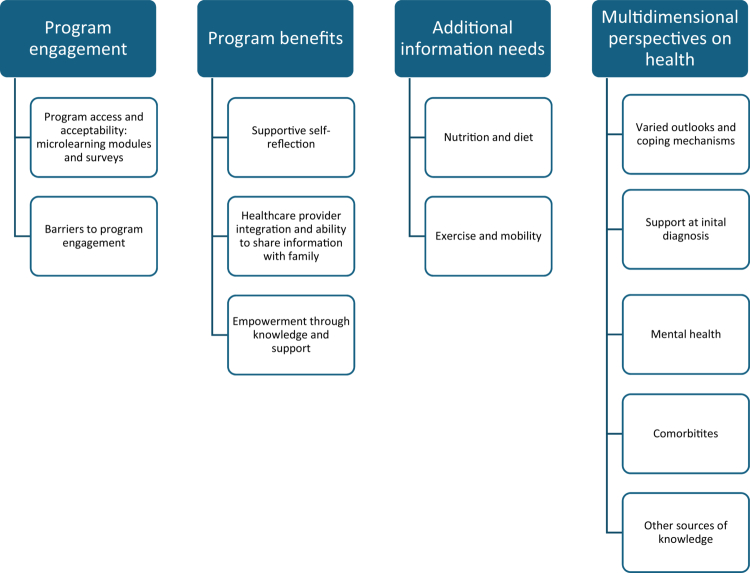
Interview themes. Diagram demonstrating four overarching themes that emerged from the interviews, with 12 subthemes under these themes. First theme: Programme engagement, subthemes: programme access and acceptability; barriers to programme engagement. Second theme: Programme benefits, subthemes: supportive self-reflection; healthcare provider integration and ability to share information with family; empowerment through knowledge and support. Third theme: Additional information needs, subthemes: nutrition and diet; exercise and mobility. Fourth theme: Multidimensional perspectives on health, subthemes: varied outlooks and coping mechanisms, support at initial diagnosis; mental health; comorbidities; other sources of knowledge.

The interviews with participants centred on three main aspects: their experiences and feedback regarding the programme, other HF-related topics they wished to learn more about (either because these topics were not covered in the programme or because they sought additional information), and their broader discussions about health. Based on the interviews, four key themes and several sub-themes were identified: programme engagement (modules and surveys); perceived benefits; additional information needs; and multidimensional perspectives on health. See Figure 2 for a summary of findings.

### 3.1. Engagement with the enhanced HF care programme

#### 3.1.1. Programme access and acceptability

Most participants reported accessing the programme. In total, 11 participants had watched the first five micro-learning modules, and 13 had completed at least one fortnightly survey monitoring depression and physical symptoms. When asked how they accessed the programme, most participants (*n* = 15) reported using their mobile phones, and 3 reported accessing it on a home computer.

Participants were asked how acceptable they found the programme, prompted with questions that examined the survey and micro-learning module ease of access, language, appropriateness, timeliness, and user-friendliness.

Most participants responded that the micro-learning modules were acceptable. Participants highlighted the convenience of having an information tool that they could access after hospital discharge, in their own time and even watch multiple times.

*Yeah. Well, a couple of them. I watched a couple of times cause I, I wanted to, I wasn't sure on just whether I got it quite right. But other than that, it was quite simple to go back and watch them, yeah.* (Interviewee 17, 1 month)

Participants described the modules as ‘straightforward,’ ‘to the point,’ and ‘didn’t use language people can’t understand’.

*Everything else was very clear, and you know I think for, um, people who have just found out that they may be in that situation, I think it was, it was very, very good.* (Interviewee 3, 1 month)

*I think if you had either medical or mental [health] problems that it would be excellent.* (Interviewee 18, 1 month.)

Participants spoke of the modules being adequately sensitive while not ‘shining over the topics.’ However, differing feedback on one module were received. One participant expressed the opinion that the module ‘How can I best plan for my future’ was not suitable for the video format; it covered advanced care planning, including wills and medical guardianship, and prognosis for patients with HF. This participant found the mortality statistics in the module distressing and spoke about it with their GP:

*It was the one about the fact that the majority of people, or 50% of the people, um who have, um who have, diagnosed with um, heart failure… don’t live past five years, I thought that was very, very confronting for people that have just been told that they may have heart failure, because since then we’ve gone on and I’ve had a few more tests and my GP and my cardiologist say I’m not in heart failure, and I, I just found that one was a little bit confronting...It would be something you need to hear from a doctor in person, not, not on a tape.* (Interviewee 3, 1 month)

Conversely, other participants spoke of this module as being beneficial, with the straightforward presentation of mortality statistics a ‘wake-up call,’ ‘brought it home’ and ‘things to consider’.

Most participants described the fortnightly surveys they received to monitor their emotional and physical health as ‘acceptable,’ ‘sensitive’, and ‘appropriate’.

*Its good quality stuff and easy to follow and easy to understand* (Interviewee 5, 1 month)

*That wasn't too hard to sort of read and follow through and that sort of stuff, so it was good.* (Interviewee 16, 1 month)

A desire for additional survey response options to accompany the validated measures was expressed by several participants.

*An optional comment field next to some of them, or even an additional comment field at the bottom where you could put extra.* (Interviewee 11, 1 month)

*There's like good and easy. There's no room for comments, but yeah, all very straightforward.* (Interviewee 4, 6 months)

#### 3.1.2. Barriers to programme engagement

Overall, participants valued the programme, but engagement was influenced by competing priorities. Participants indicated that the micro-learning modules provided broad, useful information, but some felt the content didn’t fully align with their unique circumstances or questions. Seven participants did not watch the modules, and five did not complete the surveys. When prompted, the reasons given for not using the programme stemmed from the participants not being interested in or prioritising it in their lives, either because they were too busy or time-pressed (four participants), too unwell or overwhelmed (one participant), not feeling the need (two participants) or being ‘*too lazy*’/‘*didn’t get around to it*' (two participants).

Some participants also highlighted challenges they had with accessing the programme. These included not receiving the prompting emails at all, or the emails going to their spam folder. One participant experienced technical difficulties, which were then resolved.

For participants who had accessed the micro-learning modules, a couple of participants that expressed that the topics were too broad for their specific situation:

*In my circumstance, it was a little bit too broad…but that's, that's fine. As I said, the surveys were a big benefit for me.* (Interviewee 5, 6 months)

*And although the videos give you, they let you know what's going on... It's a broad covering of everything that's going on, but there are different questions that aren't covered in the video that come up for me. So, but I thought they were pretty good.* (Interviewee 11, 1 month)

### 3.2. Benefits of the enhanced HF care programme

Participants were asked to discuss the benefits of the programme. They noted that the programme encouraged self-reflection and empowerment and was a useful tool for sharing with their support team.

#### 3.2.1. Supportive self-reflection

Participants were asked if the self-care advice given at the end of the surveys based on their survey responses was helpful, and the majority responded favourably. This theme highlights how participants found value in the surveys as a tool for personal reflection—especially around mental health. Participants suggested that the survey questions helped them reflect ‘more deeply’ on their health and emotions, especially regarding their mental health.

*Acceptable. Yes. Yes, it made me think about things. And yeah, a little more in depth, I guess. And yeah, now I'm doing quite fine in that respect. That's good. Yeah. I get a little bit anxious every now and again when I've got something on, but yeah. No, that's yeah, that's understandable.* (Interviewee 18, 1 month)

*The surveys were helpful. It just made me just sort of reflect on my situation. You know, it was a good opportunity to stop and have a look at how am I actually feeling. So the surveys I was quite happy with. I found them useful just to make me stop and go, OK. Am I stressed? No, I'm not stressed. Am I having trouble showering? Yeah, I am. all that sort of thing.* (Interviewee 5, 6 months)

#### 3.2.2. Healthcare provider integration and ability to share information with family

This theme highlights how participants found value in the surveys as a mechanism for connecting their responses to their healthcare practitioners, which they viewed positively.

*It made me stop and think about it, which also prepped me a bit for visits with the cardio specialists and so forth as well. So I understood what was going on.* (Interviewee 5, 6 months)

Participants also expressed that monitoring their mental health and having alerts sent to their general practitioner (GP) were beneficial.

*I think it's good that it goes back to your GP and then you discuss it with the GP. I think that's good.* (Interviewee 4, 1 month)

In general, participants spoke positively about having supportive care systems composed of their GPs, cardiac and other medical specialists, and hospital nurses who provided care after their HF diagnosis.

*Yeah, they're, they're good people. They're easy to speak to, and they speak to me regularly, so that's good.* (Interviewee 5, 6 months)

In this context, the programme was a helpful supplement to the information received from their medical team, while also providing them with additional topics to discuss.

Participants also provided feedback that the micro-learning modules were a useful resource for sharing with their family members, carers and anyone supporting them through their diagnosis and recovery. One participant expressed sharing the resource with their spouse and teenage children to educate them, highlighting the importance of family support systems in recovery.

*Being in hospital as well, it triggered the kids to ask a lot of questions like the youngest being 15. There he's, he had a lot of questions, and it came as a big shock to them... So, they've asked a lot of questions that I said to ‘if you want to watch videos, a video there or this is what I've taken from the video’ ... My family, I was able to sort of share it with them and, and I think that's a good thing about the programme. It's not, you watch it once and it's gone, but you can sort of forward it and if they want to watch it, you don't have to force it on them. But if they've got any questions, it's just somewhere for them to start as well.* (Interviewee 11, 1 month)

#### 3.2.3. Empowerment through knowledge and support

In general, participants said they found the programme beneficial to their recovery and felt more educated on HF.

*I thought they [modules] were all, all pretty much informative. A lot of it I had never heard of before.* (Interviewee 14, 6 months)

*I found a lot of it was very interesting and useful, which is a lot of it, sort of, I hadn't heard of before.* (Interviewee 15, 6 month)

Participants reported feeling more empowered to manage their HF diagnosis and recovery.

*There's things that you've got to change and it's, it's not a doom and gloom kind of process...It tells you what to do to manage it, and I think that's the most positive message through the videos to let, to let you know that it's manageable. And if you if you want to, I suppose, enjoy your life and live for a lot more years, then you've got to manage it.* (Interviewee 11, 1 month)

When prompted on whether any modules were most helpful, several participants highlighted the Module on symptoms being beneficial. Participants expressed having a better understanding of ‘knowing what to expect’ and ‘how some problems are intertwined with other things’.

### 3.3. Additional information needs

Participants were asked whether they felt any topics were missing from the online micro-learning modules, or if there were topics they wished to know more about. Most participants expressed that the programme was comprehensive and detailed enough for their needs. However, some participants highlighted wanting to know more details about diet and exercise that is appropriate for HF recovery.

#### 3.3.1. Nutrition and diet

Three participants expressed a desire to learn more about diet and nutrition. At the six-month mark, one participant wished they had information on ‘what is the best diet’ for living with HF. Another participant mentioned that they had received pamphlets in the hospital regarding alcohol and sodium intake. They felt that this information could have been included and expanded upon in the programme, detailing ‘what is acceptable, and what isn't?’ Another participant spoke in detail about diet and nutrition, highlighting the challenges of reducing sodium. Another participant concluded that it would be helpful if the programme provided resources for meal planning on a low-sodium diet, sodium calculators, and/or visual representations of a day's worth of low-sodium meals.

*I suppose it's just a little bit more about understanding, not making people dieticians or nutritionists, but giving them a, a bit more detail about that information panel that's on the product and why it's important…. Like they've told me … ‘cut my sodium levels back and do this, and I've got to eat healthier’. Well, what is healthy?* (Interviewee 11, 1 month)

#### 3.3.2. Exercise and mobility

The programme included a module on the importance of physical activity. Some participants suggested that the information on exercise was not detailed enough, and they wished to know what physical activities that were appropriate for HF recovery.

Another participant spoke of anxiety around returning to exercise/exertion and wished they had more information:

*I suppose something that say says “you may feel anxiety about restarting exercise…, then you know you're not alone in it. And then if I've said something after about “here's a link to the Heart Foundation” or to a particular reference material. Or “please speak to your, your physician about setting up an exercise plan” because I had no, I had no idea what to do.* (Interviewee 11, 1 month)

Five participants discussed mobility extensively throughout their interviews. They cited it as a barrier to recovery and to improving their health.

*Things seem to mount up on top of each other, and if you don’t have mobility, it stops you from doing about 99% of everything else.* (Interviewee 8, 1 month)

*You know, from time to time like I do, I do struggle with walking… It's not because of the heart failure. I don't think. I think it's more because of my age*. (Interviewee 1, 1 month)

Two participants also spoke of comorbidities presenting as barriers to exercise and suggested that the advice in the programme could be tailored around exercise while managing comorbidities.

### 3.4. Multidimensional perspectives on health

Broader themes that multiple participants raised included their views on mental health, their comorbidities, and their general outlook on their health.

#### 3.4.1. Varied outlooks and coping mechanisms

Participants expressed a range of emotional responses and outlooks during the interviews, reflecting diverse experiences and coping styles. While some conveyed a sense of optimism or acceptance, others described feeling overwhelmed and stressed, particularly in relation to the challenges of managing their diagnosis, attending appointments, and enduring extended hospital stays.

*It, as you would imagine, it gets very overwhelming when you're talking to lots and lots of specialists and medical people and you feel very unwell to start with, so you know it, it gets overwhelming.* (Interviewee 4, 6 months)

*Obviously, it’s a stressful condition to have.* (Interviewee 2, 1 month)

#### 3.4.2. Support at initial diagnosis

Some participants expressed shock at their initial diagnosis. This was caused by lack of education on HF, especially where symptoms did not match preconceived ideas about HF. With time and education, participants felt acceptance with their diagnosis.

*And when I went to the hospital, they said you had heart failure and I got a bit of a shock when they told me because I thought, I didn't know that that would happen…that's the only thing I've really I've, I've learned that heart failure is not what I thought it was.* (Interviewee 1, 1 months)

Others were more positive in tone, especially where they were acquiescent of their health diagnosis. Some participants expressed feeling ownership and responsibility for their health and recovery, and downward comparison, comparing their circumstances to other people’s, which made them better about their own situation.

*And I suppose what I mean to say is, there’s a lot of people in a lot worst situation… I don’t think I have a particularly bad one as such if you know what I mean, there are people with a lot worst.* (Interviewee 14, 1 month)

*You've gotta help yourself if you want to get out...you can't, no one else can help you get out of that, you know…you've just got to put up with these things in life, haven't you.* (Interviewee 17, 1 month)

#### 3.4.3. Mental health

Participants were asked about their mental health, with an emphasis on depression and anxiety. Participants found the modules on mental health acceptable and helpful for reflecting on their wellbeing.

*I was, I think I read them and thinking “did I have depression, did I have this and that?” But I think I was just, um, down because no one seemed to be getting on top what was wrong with me, but once that was all worked out, everything was fine, so I think anyone, anyone has those feelings when you’re not getting well and you think you should be.* (Interviewee 3, 6 months)

*And like talking about slowing your breathing down and holding your belly and... that’s really good.* (Interviewee 4, 6 months)

Some participants distanced themselves from depression and anxiety, seeing poor mental health as something that affects other people rather than themselves.

*Yes, as far as that, I never get depressed. I, I've never had depression. Oh, you know you get sad, but that's, that's not depression ... and, and I've never. I've never suffered in that way at all. You know, I'm very, I'm a very positive, a very positive person… It's a really terrible thing to go through. I think, you know, because it's hard, it's hard to get over...yeah. But I'm, I'm OK that way.* (Interviewee 1, 6 months)

*So that’s why I think like I haven’t thought too much about, you know like I’ve read all the modules about, you now the mental health and da da da da, look I don’t really care too much about that... I might be a little bit odd and different, but first of all, there’s nothing you can do about it.* (Interviewee 14, 6 months)

For other participants, they could recognise how their diagnosis was impacting their mood and mental health.

*Getting to know that you've gotta help yourself if you want to get out.* (Interviewee 17, 1 month)

#### 3.4.4. Comorbidities

Some participants framed their HF in the context of having comorbidities. Participants described managing Parkinson’s Disease, type 2 diabetes mellitus, cellulitis, gout, osteoarthritis and clinical depression, as well as being treated for ‘kidney issues’ and ‘issues with bowels’. Comorbidities meant more touchpoints with the health system through specialist and GP appointments, which allowed participants opportunities to learn about their conditions.

*Well I’m still getting calls, well having to do, uh, regular communications with um, the specialists because um, I have a cardio, a haematology and a lung specialist as well as my regular GP [laughs], so between the four of them, I’m pretty well covered.* (Interviewee 5, 1 month)

Having comorbidities made participants feel more overwhelmed. Some participants mentioned that the information in the learning modules was not specific enough or helpful for their situation.

Three participants raised COVID-19, describing it as a disruptor to their recovery. They perceived that catching COVID-19 either triggered their HF symptoms or helped identify their HF more clearly.

*And I go regularly to, to my GP and I just keep well, you know, for diabetes and also for, for my heart and cholesterol. You know, I've got a few, I've got a few problems now [laughs] in this, in this last 12 months. But it really all started with COVID because I got COVID about 18 months ago... and after that, before that, I was quite healthy. And after the COVID and they told me that I've got long COVID because even my voice hasn't come back to what it used to be.* (Interviewee 1, 1 month)

#### 3.4.5. Other sources of knowledge

Participants were asked whether they accessed other sources of information for HF. Most participants reported receiving booklets and pamphlets while in hospital, which was their main source of information on HF.

*I got um, leaflets on heart failure when um, the nurses came around. When I came home from hospital, they brought- they brought all of that, and I’ve read all through it. You know, so I understand it, I understand it now.* (Interviewee 1, 1 month)

Some participants said having multiple touchpoints with the health system meant they were informed and had plenty of opportunities to learn about HF:

*I've got a wonderful GP and, and I, I know I can ask my, my cardiologist anything as well. So yeah, now I've got a pretty good support system going on. Yep.* (Interviewee 18, 1 month)

Participants had mixed reactions to whether they searched the internet for information of HF. Some expressed using internet searches about HF, and considered being able to search their conditions online was helpful. However, others avoided searching the internet, regarding the information unhelpful, or a cause of confusion and further anxiety.

*Oh no I find Dr Google’s not that good [Laughs].* (Interviewee 14, 1 month)

*I mean everybody, I guess, everybody Dr Googles themselves these days.* (Interviewee 16, 1 month)

## 4. Discussion

This research sought to explore participants' experiences with the Enhanced HF Care programme. Their feedback provided an understanding of how participants engaged with the programme, perceived its value, and identified areas for improvement. Overall, participants valued the programme for its educational benefits and support in managing their health, particularly the ability to access the content at a convenient time following hospital discharge and integrate it into their ongoing care.

However, engagement was variably affected by competing priorities and information load, elaborated in Section 4.2–4.3. Addressing missing topics and enhancing initial support could improve programme relevance and uptake.

### 4.1. Patient empowerment through knowledge and support

Participants reported significant benefits from the programme, including improved understanding of their condition, enhanced ability to reflect on health, and increased confidence in sharing information with family and healthcare providers. These findings illustrate several mechanisms through which the programme fostered empowerment. For example, the structured educational materials enabled repetition and consolidation of key concepts, which helped participants obtain practical knowledge they felt capable of applying in daily life. The prompts within the programme encouraged ongoing reflection on symptoms and behaviours, supporting participants to make clearer connections between their actions and their health status. This reflective process appears to have strengthened their sense of control and agency. By equipping participants with accessible information, the programme improved their capacity to communicate with family members and clinicians, supporting a more informed understanding of their health, and reinforced their role as active partners in care.

Participant reports of improved symptom recognition further suggest that repeated exposure to information and the opportunity to practice self-monitoring enhanced their confidence in recognising symptoms. Their descriptions of the care team as a trusted source of knowledge point to another mechanism of empowerment: consistent guidance and support, which can reduce uncertainty and encourage engagement.

These combined mechanisms—repetition, reflection, and supported communication underscore the programme’s potential to empower patients through education and self-management tools. Patient empowerment—a patient’s ability to control their health and be involved in their care—has been associated with better health outcomes including health-related quality of life (Jonkman et al., 2016; Pekonen et al., 2020; Sethares et al., 2014) and reduced all-cause hospital readmissions and readmissions due to HF (Jovicic et al., 2006). It is considered an essential feature of high-performing health services (Katz et al., 2020; Marzban et al., 2022). Many participants framed their recovery as their own responsibility and spoke of the programme as an opportunity for them to take steps to improve and manage their health, indicating a shift from passive receipt of care to active engagement.

The ability to share information with family and clinicians suggests that programmes can strengthen social support networks and improve continuity of care. These benefits suggest that educational components and links to care teams are central to fostering engagement and self-management. The theme Empowerment Through Knowledge and Support reflects findings from studies emphasising the role of digital health in fostering patient activation and self-management. The programme’s participants’ appreciation for the ability to share information with family and clinicians aligns with other research showing that digital platforms strengthen social support networks and continuity of care (Zhai et al., 2023).

### 4.2. Programme engagement

Despite positive feedback, barriers to programme engagement emerged as a sub-theme. Seven participants did not view any modules, and five did not complete any of the fortnightly monitoring surveys. Uptake was limited by other priorities and information overload. This pattern mirrors broader digital health findings on barriers to uptake and engagement, such as competing priorities, information overload, and usability (Batterham et al., 2021; Ross et al., 2023).

This could be overcome by promoting the usefulness and ease of integration into daily routines, which research has found can influence engagement (Hu & Xiao, 2025). The final uptake of the programme by all intervention participants will be presented upon completion of the study, however, the high uptake among interviewees indicates a general readiness to engage with online health interventions.

### 4.3. Dealing with information overload: flexibility in timing

The amount of information provided after HF hospitalisation can be overwhelming for patients (Hestevik et al., 2019). However, the flexibility offered by digital health programmes can be beneficial. Participants reported that the online format of the programme allowed them to revisit the modules multiple times, enabling them to absorb the information at their own pace. This repeated exposure can lead to better understanding and management of their HF (Deshpande et al., 2023; Hwang et al., 2020; Strömberg et al., 2006). Thus, pacing and repeat access may partially offset information overload in the immediate post-discharge period.

### 4.4. Support at initial diagnosis and additional recommendations

Shock at initial diagnosis was identified during this study, aligning with experiences described in other patients in medical literature (Olano-Lizarraga et al., 2016). Patients recently diagnosed with HF might not fully understand the condition, why it developed, or its long-term implications (Rodriguez et al., 2008). Providing additional support at the point of diagnosis may alleviate the confusion and distress experienced by newly diagnosed patients. Participants viewed the programme as a helpful supplementary tool. Participants identified some gaps in content, particularly around nutrition, diet, exercise, and mobility. Addressing these gaps could enhance relevance and engagement, particularly for participants seeking actionable strategies. Further information for HF patients could include practical advice on nutrition, exercise goals, and suggestions for accessing cardiac rehabilitation.

Overall, participants’ testimonies highlight the programme's value and reinforce its potential to complement traditional healthcare services by providing ongoing support and education beyond the clinical setting. Importantly, this study extends existing digital HF literature by providing new, qualitative evidence on patient experiences with a post‑discharge digital support programme for acute decompensated HF—an area where patient perspectives remain limited. A distinctive contribution of this study is its exploration of routine mental health monitoring within a HF intervention; to our knowledge, no previous digital HF programmes have incorporated regular psychological symptom surveys as part of follow‑up. The acceptability of this feature among those who completed the surveys highlights an emerging opportunity to integrate mental health assessment into HF care pathways.

By highlighting how patients engage with educational content, symptoms monitoring, and communication features after hospital discharge, this study deepens understanding of the mechanisms that shape patient engagement in digital HF care. These insights can inform the development of future interventions that are more responsive to patient priorities, more responsive to psychosocial needs, and equitable digital health interventions.

### 4.5. Future directions

Online health programmes have the potential to play a significant role within the broader Australian public health system. Programmes like Enhanced HF Care offer a scalable way to deliver health education and support to more patients across various clinical settings. Based on these interviews, future work should continue emphasising educational content and practical tools for self-management including symptom recognition. The findings suggest that while the programme is valued for its educational benefits and support, engagement is hindered by competing priorities and information overload. Participants reflected on broader health issues, including comorbidities, emotional wellbeing, and the need for support at initial diagnosis. Many expressed a positive outlook, characterised by ownership of illness and downward comparison, which helped maintain resilience. Mood changes were also noted, emphasising the psychological dimension of chronic illness. These reflections reveal the complexity of living with illness and the interplay between physical and emotional health. Programmes that acknowledge these dimensions can foster coping strategies and improve overall wellbeing.

### 4.6. Strengths and limitations

Most participants in the substudy and trial at this point were English-speaking men. As a result, the generalisability of these findings may be constrained by gender differences among patients with HF (Sawatari & Niitani, 2025). For example, prior research indicates women with heart failure often report significantly higher levels of anxiety and depression, compared with men (Sawatari & Niitani, 2025). Nonetheless, the final sample size was sufficient to achieve data saturation of themes. Purposive sampling was undertaken as its widespread use in qualitative research is well established (Palinkas et al., 2015). The principle of data saturation was used to ensure the adequacy of the sample (Hennink & Kaiser, 2022).

Additional limitations include that some rural communities lack adequate and reliable internet access. Since internet access was a patient inclusion criterion for this study, these concerns were not reflected in participants' responses. For these population groups, digital health interventions may present significant acceptability and usability challenges. Alternative or hybrid support methods—such as telephone-based check-ins, offline materials, or community-based support—should therefore be considered to ensure equitable access and to avoid exacerbating existing digital disparities.

## 5. Conclusion

This study offers valuable insights into caring for patients discharged from hospital with HF. Specifically, the results indicated that the online Enhanced HF Care programme is easy to understand, simple, and empowering for managing their recovery. Key aspects of the programme seen as beneficial included the ability to revisit the programme at their own pace and to share it with support persons and the care team. Future post-discharge interventions from heart failure could be enhanced by digital health solutions that purposefully integrate educational content with mechanisms for ongoing symptom monitoring.

## Supplementary Material

WETZLER 2025_COREQ Checklist .docx WETZLER 2025_COREQ Checklist .docx

## Data Availability

Deidentified data may be made available upon reasonable request.

## Clinical trial registration

The trial was registered with the Australian New Zealand Clinical Trials Registry (ANZCTR): 12622001289707.
